# Identification of serum IFN-α and IL-33 as novel biomarkers for type 1 autoimmune pancreatitis and IgG4-related disease

**DOI:** 10.1038/s41598-020-71848-4

**Published:** 2020-09-16

**Authors:** Kosuke Minaga, Tomohiro Watanabe, Akane Hara, Ken Kamata, Shunsuke Omoto, Atsushi Nakai, Yasuo Otsuka, Ikue Sekai, Tomoe Yoshikawa, Kentaro Yamao, Mamoru Takenaka, Yasutaka Chiba, Masatoshi Kudo

**Affiliations:** 1 Department of Gastroenterology and Hepatology, Kindai University Faculty of Medicine, 377-2 Ohno-Higashi, Osaka-Sayama, Osaka 589-8511 Japan; 2 Clinical Research Center, Kindai University Hospital, Osaka-Sayama, Osaka Japan

**Keywords:** Biomarkers, Gastroenterology, Pancreatitis, Autoimmune diseases

## Abstract

IgG4-related disease (IgG4-RD) is a multi-organ autoimmune disease characterized by elevated serum IgG4 concentration. Although serum IgG4 concentration is widely used as a biomarker for IgG4-RD and type 1 autoimmune pancreatitis (AIP), a pancreatic manifestation of IgG4-RD, a significant number of patients have normal serum IgG4 levels, even in the active phase of the disease. Recently, we reported that the development of experimental AIP and human type 1 AIP is associated with increased expression of IFN-α and IL-33 in the pancreas. In this study, we assessed the utility of serum IFN-α and IL-33 levels as biomarkers for type 1 AIP and IgG4-RD. Serum IFN-α and IL-33 concentrations in patients who met the diagnostic criteria for definite type 1 AIP and/or IgG4-RD were significantly higher than in those with chronic pancreatitis or in healthy controls. Strong correlations between serum IFN-α, IL-33, and IgG4 concentrations were observed. Diagnostic performance of serum IFN-α and IL-33 concentrations as markers of type 1 AIP and/or IgG4-RD was comparable to that of serum IgG4 concentration, as calculated by the receiver operating characteristic curve analysis. Induction of remission by prednisolone treatment markedly decreased the serum concentration of these cytokines. We conclude that serum IFN-α and IL-33 concentrations can be useful as biomarkers for type 1 AIP and IgG4-RD.

## Introduction

IgG4-related disease (IgG4-RD) is a recently discovered autoimmune disorder characterized by elevated IgG4 serum levels and by the accumulation of IgG4-expressing plasmacytes in the affected organs^1–3^. This autoimmune disorder is also characterized by multiple organ involvement^1–3^. The clinicopathological features of IgG4-RD were first identified in patients with autoimmune pancreatitis (AIP) and then, various kinds of autoimmune diseases formerly classified as AIP, autoimmune sialadenitis, autoimmune sclerosing cholangitis, and retroperitoneal fibrosis, have become recognized as organ-specific manifestations of systemic IgG4-RD^1–4^. Now AIP is classified into type 1 and type 2 AIP, the former being the pancreatic manifestation of IgG4-RD^5^.

Elevated IgG4 serum levels are a diagnostic marker for type 1 AIP and IgG4-RD^6,7^. Moreover, serum IgG4 concentration over 280 mg/dL is associated with multiple organ involvement and the risk of relapse^8,9^. Thus, the measurement of serum IgG4 concentration is useful not only for the diagnosis but also for the evaluation of disease severity in IgG4-RD. It should be noted, however, that the levels of this particular IgG subtype are not always useful for the differential diagnosis or evaluation of disease severity in IgG4-RD and type 1 AIP. About 20% of patients with type 1 AIP have normal levels of IgG4 in the serum^10^. Furthermore, serum IgG4 concentrations are sometimes elevated in patients with pancreatic cancer^11^. In addition, disease flare-ups are seen in about 10% of patients with type 1 AIP even in the presence of normal IgG4 levels^12^. Therefore, it is necessary to develop novel biomarkers that could be useful for the diagnosis and disease progression monitoring of type 1 AIP and IgG4-RD.

Cytokines and chemokines serum levels may also serve as biomarkers in several autoimmune diseases. For example, serum concentrations of IL-6 and thymus and activation-regulated chemokine are well-established biomarkers of multicentric Castleman’s disease (MCD) and atopic dermatitis, respectively^13,14^. Recently, we reported that the production of interferon (IFN)-α and interleukin (IL)-33 by plasmacytoid dendritic cells (pDCs) mediates chronic fibroinflammatory responses in experimental AIP and human type 1 AIP^1,15–19^. Our previous studies provided evidence that pancreatic accumulation of pDCs, which may produce large amounts of IFN-α and IL-33, underlies the immunopathogenesis of type 1 AIP and IgG4-RD^1,15–19^. Furthermore, we had a case of type 1 AIP and IgG4-RD in which the serum concentrations of IFN-α and IL-33, rather than the serum concentration of IgG4, were highly associated with disease activity^20^. Based on our previous data, in the present study, we tested the hypothesis that serum concentrations of IFN-α and IL-33 could be useful biomarkers for type 1 AIP and IgG4-RD.

## Methods

### Study subjects

To validate the utility of serum IFN-α and IL-33 concentrations as biomarkers of type 1 AIP and IgG4-RD, we enrolled 21 consecutive, newly diagnosed patients who visited the Kindai University Hospital between June 2018 and December 2019 and met the diagnostic criteria for definite type 1 AIP^7^ and/or IgG4-RD^6^.Twelve patients met the diagnostic criteria for both IgG4-RD and type 1 AIP. Two patients met the criteria for IgG4-RD alone whereas seven patients met that for type 1 AIP alone. The 21 patients that fulfilled the diagnostic criteria for definite type 1 AIP and/or IgG4-RD are thereafter called type 1 AIP/IgG4-RD patients in this manuscript. Histological specimens (n = 18) were obtained by endoscopic ultrasound-guided fine-needle aspiration in 16 patients, endoscopic retrograde cholangiography-guided biopsy in one patient, and surgical resection in one patient, and analyzed by a pathologist. Histologic analysis was not performed for the three patients that were diagnosed with type 1 AIP based on typical radiographic findings and serum IgG4 concentrations^7^. Positron emission tomography/computed tomography (PET/CT) using ^18^F-2-fluoro-2-deoxy-D-glucose was performed in 19 patients (90.5%) for the diagnosis and evaluation of disease activity^21^. The number of affected organs was determined by PET/CT in 19 patients and contrast-enhanced whole-body CT in two patients. IgG4-RD responder index (IgG4-RD RI) was calculated as previously described^22^.

Twelve chronic pancreatitis (CP) patients who met diagnostic criteria for definite alcoholic CP were also enrolled as the disease control group^23^. We selected typical CP patients exhibiting pancreatic stones and main pancreatic duct dilatation, as per CT examinations.

Serum samples were obtained at the diagnosis of type 1 AIP/IgG4-RD or CP. In addition, serum samples were also obtained from eight healthy control (HC) cases. The study protocol conformed to the ethical guidelines for human clinical research established by the Japanese Ministry of Health, Labor and Welfare and was approved by the ethics committee of the Kindai University, Faculty of Medicine. Written informed consent was obtained from all patients and healthy controls at enrollment.

### Prednisolone treatment

Twelve patients with type 1 AIP/IgG4-RD received oral administration of prednisolone (PSL) at the initial dose of 0.6–0.7 mg/kg for the first two weeks followed by the scheduled tapering regimen (5 mg every two weeks). In all of these 12 patients, PET-CT confirmed improved disease activity at the time of serum sampling after PSL administration (at the remission state, around 37 weeks after the initiation of PSL treatment). However, serum samples were not collected from the remaining nine patients (of the 21 type 1 AIP/IgG4-RD patients), since six months had not passed after the initiation of PSL treatment.

### Serum Ig subtype analysis

Serum concentrations of total IgG, IgG4, IgA, and IgE were measured as described previously^24^. Briefly, IgG and IgM serum concentrations were measured using a turbidimetric immunoassay and that of IgE were measured using a fluorescence enzyme immunoassay. Serum levels of IgG4 (normal range 11–121 mg/dL) were measured using a latex immunoturbidimetric method. Serum concentrations of IgG1, IgG2, and IgG3 were determined using enzyme-linked immunosorbent assay (ELISA) commercial kits, as described previously (Thermo Fisher Scientific, Waltham, MA)^25^. Serum IgG subtypes’ concentrations in the HCs analyzed in this study ranged as follows: IgG1 (245.5–1129.1 mg/dL), IgG2 (141.4–948.6 mg/dL), and IgG3 (17.1–150.9 mg/dL).

### Serum cytokine analysis

Serum concentrations of IFN-α and IL-33 were measured using commercial ELISA kits from R&D systems (Minneapolis, MN)^20,24^. Serum concentrations of IL-1β, IL-6, and TNF-α were determined using other commercial ELISA kits, as previously described (Thermo Fisher Scientific)^26^.

### Serum markers diagnostic performance

Diagnostic performance of serum markers (IgG, IgG4, IFN-α, and IL-33) was assessed by receiver operating characteristic (ROC) curve analysis; the area under the curve (AUC) values for each biomarker were calculated. The optimal cut-off points of serum markers were determined based on the minimum values of (1 − sensitivity)^2^ + (1 − specificity)^2^.

### Statistical analysis

Baseline characteristics are indicated as percentages for categorical variables or means ± standard deviation for continuous variables. The Mann–Whitney U test, which is a nonparametric version of unpaired t test, was used to evaluate the differences between two groups for baseline characteristics and serum markers. The Kruskal–Wallis test, which is a nonparametric version of one-way ANOVA, was used to evaluate the differences between more than two groups for serum markers. For post hoc analysis (after the Kruskal–Wallis test), the Bonferroni corrected Mann–Whitney U test was performed for comparison between two groups, where each *P*-value was derived by multiplying a *P*-value derived by the number of tests. The Wilcoxon signed rank test, which is a nonparametric version of paired t test, was used to compare serum markers before PSL treatment to those at maintenance treatment. The Spearman correlation coefficient was used to evaluate the association between the different serum markers evaluated in this study. All statistical analyses were performed using the GraphPad Prism 6.0 software (GraphPad Software Inc., La Jolla, CA). *P*-values < 0.05 indicated statistical significance.

## Results

### Patient profiles

A total of 21 type 1 AIP/IgG4-RD patients that fulfilled the diagnostic criteria for definite type 1 AIP^7^ and/or IgG4-RD^6^ were enrolled in this study. Twelve CP patients met the diagnostic criteria for definite CP^23^. The characteristics of patients with type 1 AIP/IgG4-RD and CP are shown in Table 1. No significant differences were seen with respect to serum biochemistry parameters or tumor markers between CP and type 1 AIP/IgG4-RD patients.

**Table 1:** Clinical characteristics of the patients with type 1 autoimmune pancreatitis/IgG4-related disease and chronic pancreatitis.

Variable	Type 1 AIP/IgG4-RD (n = 21)	CP (n = 12)	*P* value
Age	67.5 ± 2.3	67.6 ± 3.5	0.919
Gender, male, n (%)	16 (76.2%)	9 (75%)	1.000
Diabetes mellitus	9 (42.9%)	8 (66.7%)	0.282
Other comorbidities	Asthma (3), RA (1)	–	-
**Laboratory data**			
WBC, /µL	6577 ± 1901	5298 ± 1333	0.043
CRP, mg/dL	0.32 ± 0.40	0.45 ± 0.81	0.257
Alb, g/dL	3.8 ± 0.34	3.9 ± 0.54	0.522
AMY, U/L	98.5 ± 81.2	139.8 ± 103.1	0.124
CEA, ng/mL	3.2 ± 1.75	3.8 ± 2.32	0.564
CA19-9, U/mL	29.7 ± 33.7	36.0 ± 45.5	0.888
IgG4-RD RI score	12.1 ± 2.9		
**Organ involvement, n (%)**			
Pancreas	19 (90.5%)	–	
Bile duct	4 (19.0%)	–	
Gallbladder	1 (4.8%)	–	
Submandibular gland	6 (28.6%)	–	
Parotid gland	4 (19.0%)	–	
Lacrimal gland	2 (9.5%)	–	
Thyroid	1 (4.8%)	–	
Lymph node	15 (71.4%)	–	
Aorta/artery	5 (23.8%)	–	
Retroperitoneum	2 (9.5%)	–	
Lung	1 (4.8%)	–	
Kidney	3 (14.3%)	–	
**Number of organs involved, n (%)**			
1	2 (9.5%)		
2	6 (28.6%)		
3	6 (28.6%)		
4	5 (23.8%)		
5	1 (4.8%)		
6	1 (4.8%)		

Continuous data are presented as the mean ± standard deviation.

RA, Rheumatoid arthritis; WBC, White blood cells; CRP, C-reactive protein; Alb, Albumin; AMY, Amylase; CEA, Carcinoembryonic antigen; CA19-9, Carbohydrate antigen 19–9; RI, Responder Index.

### Profiles of serum immunoglobulins, including IgG subtypes in patients with type 1 AIP/IgG4-RD

**Figure 1:**
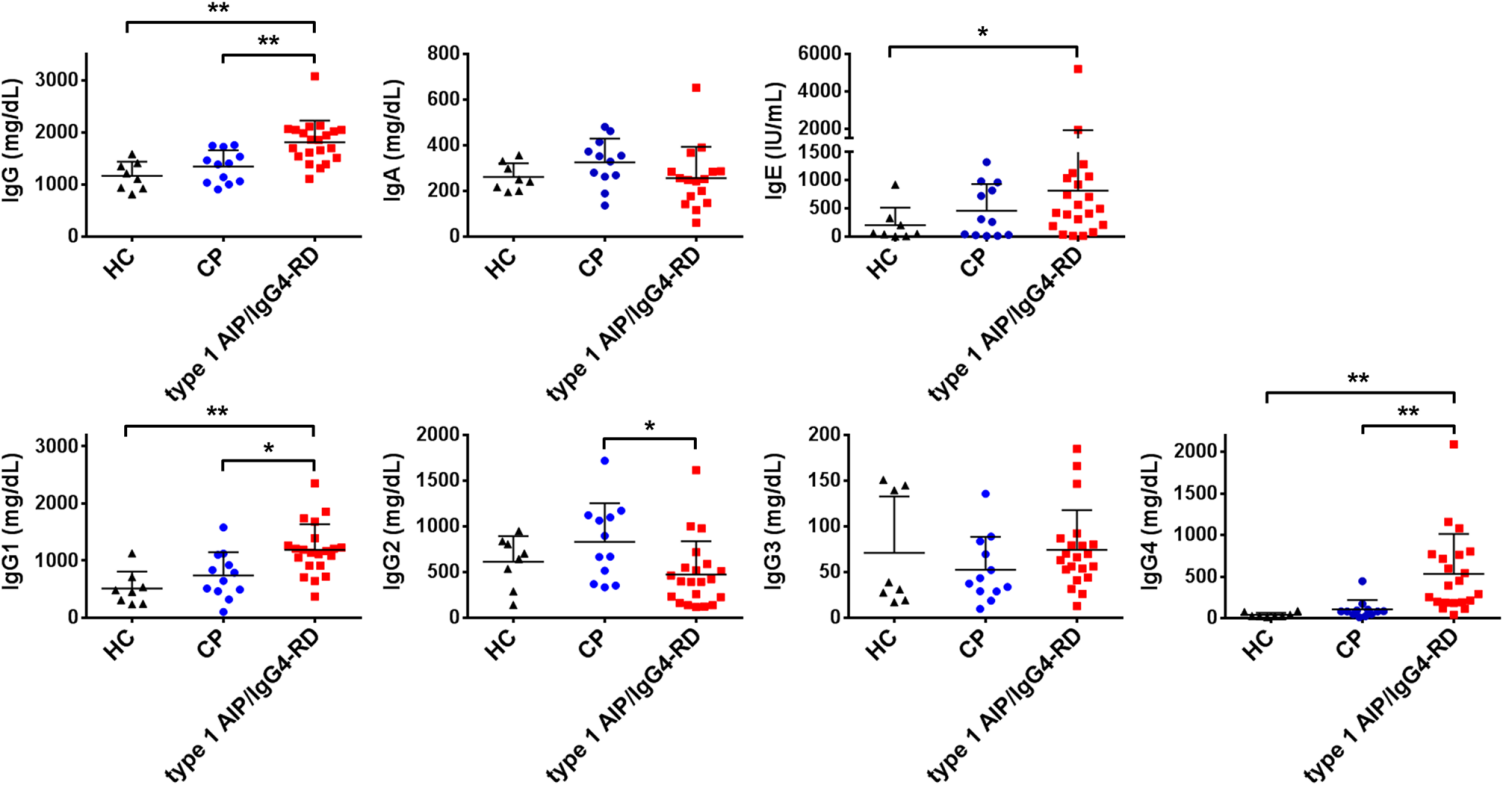
Serum concentrations of Ig and IgG subtypes in patients with type 1 autoimmune pancreatitis/IgG4-related disease and chronic pancreatitis. Serum concentrations of total IgG, IgA, IgE, IgG1, IgG2, IgG3, and IgG4 were measured in 21 patients with type 1 autoimmune pancreatitis (AIP)/IgG4-related disease (IgG4-RD) and 12 patients with chronic pancreatitis (CP), and 8 healthy controls (HCs). Each dot represents one patient. The larger horizontal line indicates the mean value and the error bar above shows the standard deviation of the mean. Significant differences between groups were determined by the Kruskal–Wallis and post hoc Bonferroni corrected Mann–Whitney U tests: *P < 0.05, **P < 0.01.

We initially tried to determine the levels of total IgG in patients with type 1 AIP/IgG4-RD. Consistent with the previous reports^4,27^, serum levels of total IgG were significantly higher in patients with type 1 AIP/IgG4-RD than in CP patients or HCs (Fig. 1 and Supplementary Table S1). Although serum concentrations of IgA in type 1 AIP/IgG4-RD patients were lower than those in CP patients, this difference was not statistically significant (Fig. 1 and Supplementary Table S1). Serum concentrations of IgE were nominally higher in type 1 AIP/IgG4-RD patients than in CP patients, but this difference was again not statistically significant (Fig. 1 and Supplementary Table S1).

We then turned our attention to the serum concentrations of IgG subtypes in patients with type 1 AIP/IgG4-RD. In line with the previous reports^4,9,27–29^, serum levels of IgG4 were much higher in type 1 AIP/IgG4-RD patients than in CP patients and HCs. In fact, IgG4 serum levels were at least three times higher in around 50% (11/21) of patients with type 1 AIP/IgG4-RD, as compared to its upper limits. In addition to IgG4, patients with type 1 AIP/IgG4-RD exhibited elevated serum IgG1 levels as compared to those in CP patients and HCs. In contrast, serum concentrations of IgG3 were comparable between patients with type 1 AIP/IgG4-RD, CP patients and HCs. Last but not least, patients with type 1 AIP/IgG4-RD exhibited lower serum IgG2 levels than those from CP patients. Thus, these data suggest that patients with type 1 AIP and IgG4-RD are characterized by elevated concentrations of serum total IgG, IgG1, and IgG4.

### Profiles of innate immunity-related cytokines in patients with type 1 AIP/IgG4-RD

**Figure 2:**
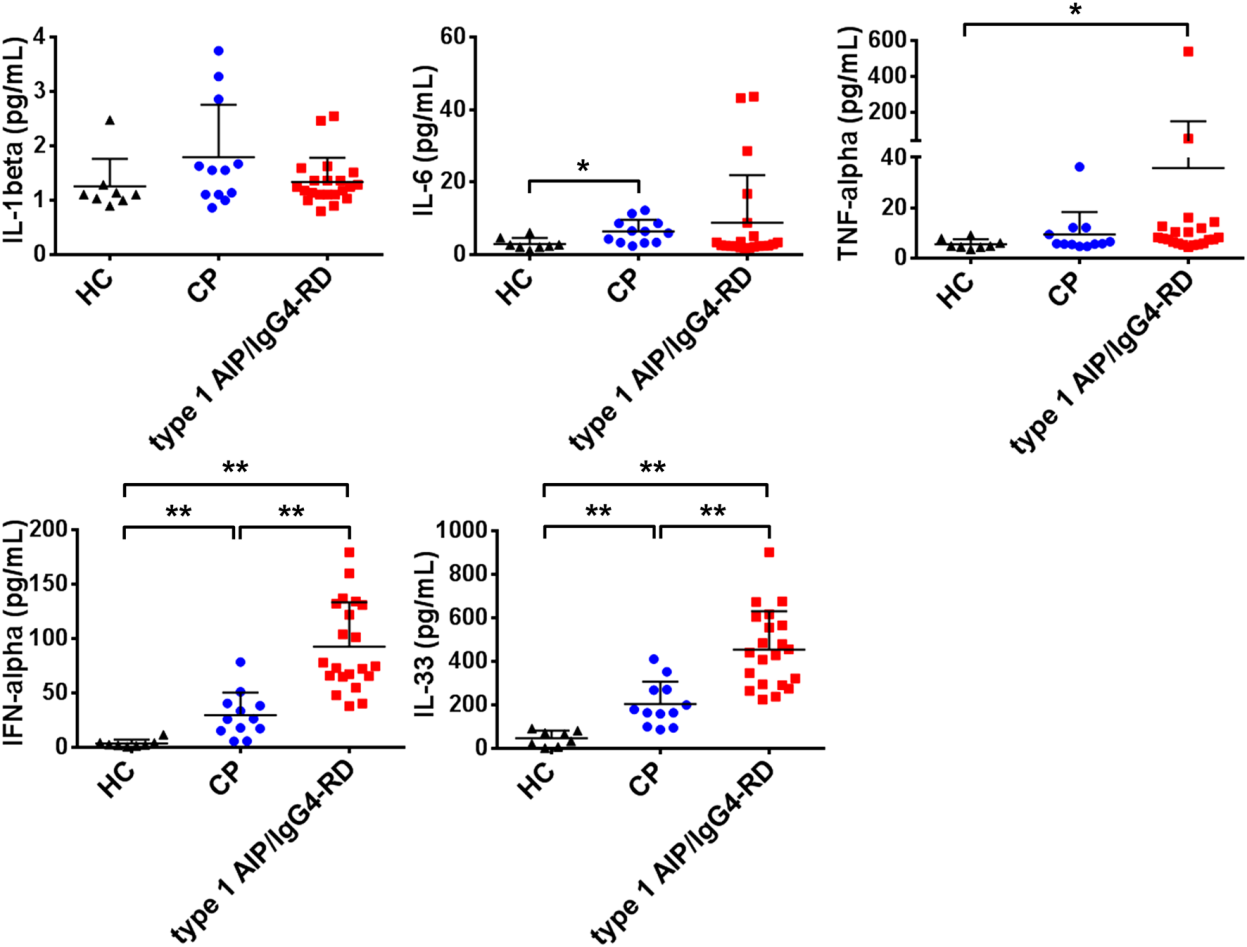
Serum concentrations of innate immunity-related cytokines in patients with type 1 autoimmune pancreatitis/IgG4-related disease and chronic pancreatitis. Serum concentrations of IL-1β, IL-6, TNF-α, IFN-α, and IL-33 were measured in 21 patients with type 1 autoimmune pancreatitis (AIP)/IgG4-related disease (IgG4-RD), 12 patients with chronic pancreatitis (CP), and 8 healthy controls (HCs). Each dot represents one subject. The larger horizontal line indicates the mean value and the error bar above shows the standard deviation of the mean. Significant differences between groups were determined by the Kruskal–Wallis and post hoc Bonferroni corrected Mann–Whitney U tests: *P < 0.05, **P < 0.01.

We have previously reported that peripheral blood monocytes and pDCs isolated from patients with type 1 AIP/IgG4-RD promoted the production of IgG1 and IgG4 by B cells from HCs in the absence of T cells^15,25^. Indeed, peripheral blood pDCs from type 1 AIP/IgG4-RD patients enhanced IgG4 production by healthy B cells in a T cell-independent and IFN-α-dependent manner^15^. More importantly, increased production of IFN-α and IL-33 by pDCs mediated chronic fibroinflammatory responses in both experimental AIP and human type 1 AIP^1,15–19^. Thus, T cell-independent cytokines produced by antigen-presenting cells, including pDCs, may be involved in the elevation of serum IgG1 and IgG4 concentrations in patients with type 1 AIP/IgG4-RD. Based on these findings from our previous reports, we evaluated the profiles of cytokines involved in innate rather than adaptive responses in this study. As shown in Fig. 2, serum concentrations of prototypical pro-inflammatory cytokines such as IL-1β, IL-6, and TNF-α were comparable among CP and type 1 AIP/IgG4-RD patients except for one type 1 AIP case that exhibited a remarkably high concentration of TNF-α. In contrast, markedly higher concentrations of IFN-α and IL-33 were seen in patients with type 1 AIP/IgG4-RD as compared to those in CP patients. Thus, these serum cytokine assays revealed that patients with type 1 AIP and IgG4-RD were characterized by elevated serum IFN-α and IL-33 concentrations. These serum cytokine data were fully consistent with our recent studies in which pDC-mediated production of IFN-α and IL-33 was shown to play a pathogenic role in the development of experimental AIP and human type 1 AIP^1,15–19^.

### Correlation between serum cytokine and IgG levels

**Figure 3:**
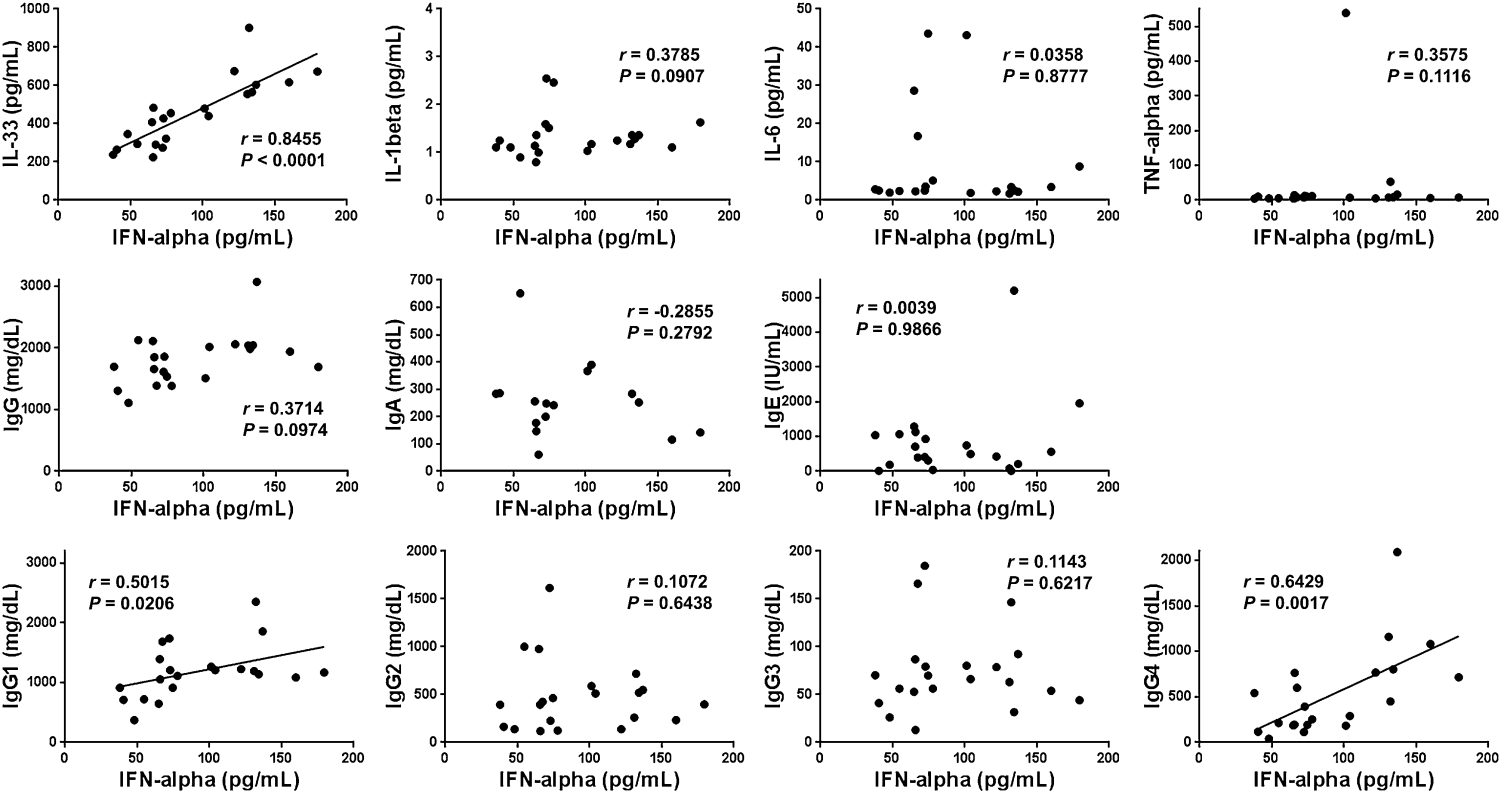
Correlation between serum IFN-α levels and those of other cytokines and Ig subtypes in patients with type 1 autoimmune pancreatitis/IgG4-related disease. Serum concentrations of IL-1β, IL-6, TNF-α, IL-33, IgG, IgA, IgE, IgG1, IgG2, IgG3, and IgG4 were measured in 21 patients with type 1 autoimmune pancreatitis/IgG4-related disease, and correlated with those of IFN-α. Each dot represents one patient. *P*-values and correlation coefficient (*r*) values, as determined by Spearman’s rank correlation test, are shown.

A strong positive correlation between the serum concentrations of IFN-α and IL-33 was seen in patients with type 1 AIP/IgG4-RD (Fig. 3). A significant positive correlation between the serum concentrations of IFN-α and IgG1 or IgG4 was also seen in type 1 AIP/IgG4-RD patients (Fig. 3). A positive correlation between IFN-α and total IgG levels was also apparent, although it was not significant. It should be noted, however, that the strength of the correlation between IFN-α and IgG4 levels was stronger than that between IFN-α and the IgG1 levels, as per the Spearman’s correlation coefficient values. Furthermore, no significant correlation between IFN-α and IL-1β, IL-6, TNF-α, IgA, IgE, IgG2, or IgG3 was observed.

**Figure 4:**
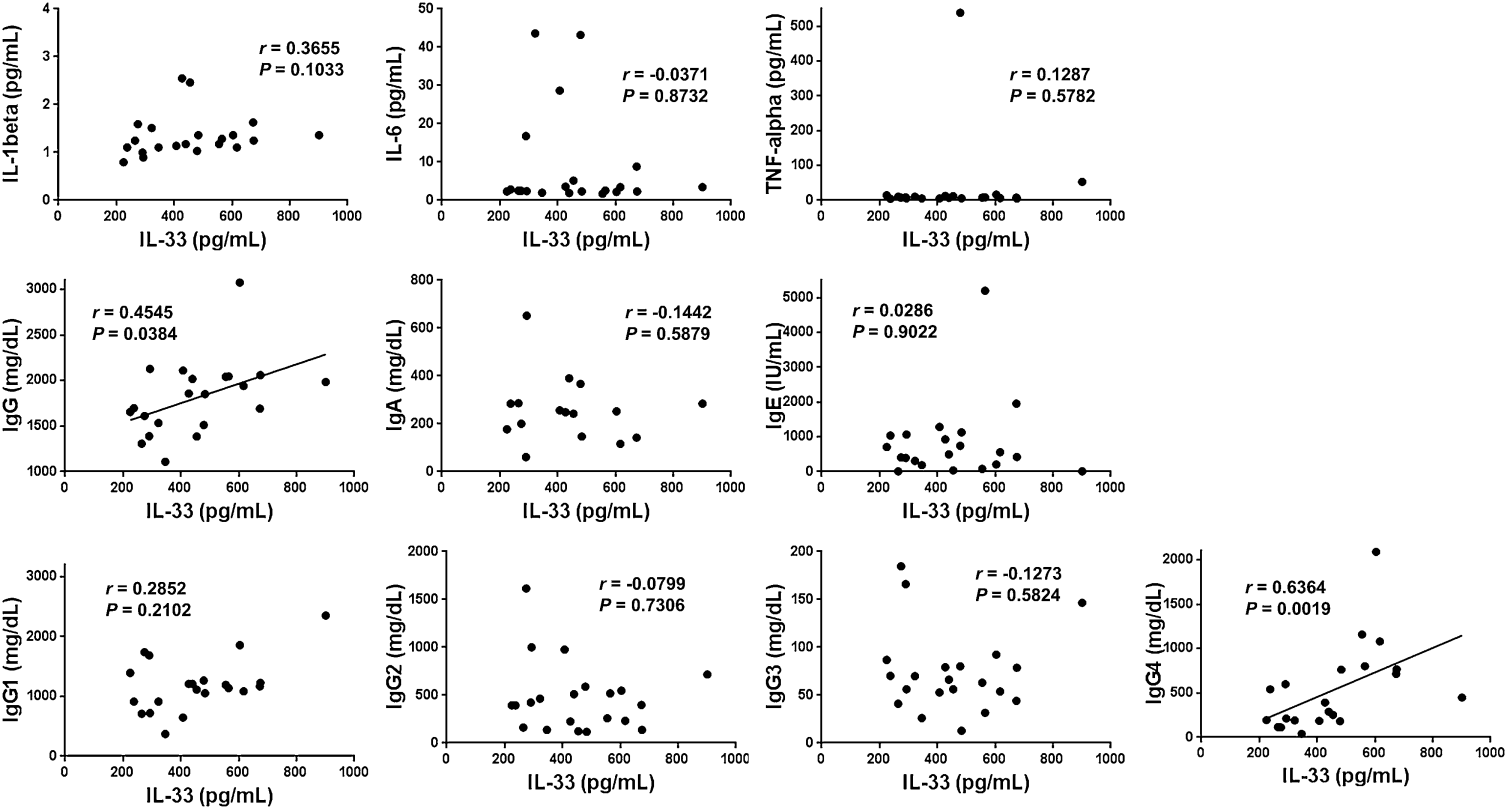
Correlation between serum IL-33 levels and those of other cytokines and Ig subtypes in patients with type 1 autoimmune pancreatitis/IgG4-related disease. Serum concentrations of IL-1β, IL-6, TNF-α, IFN-α, IgG, IgA, IgE, IgG1, IgG2, IgG3, and IgG4 were measured in 21 patients with type 1 autoimmune pancreatitis/IgG4-related disease, and correlated with those of IL-33. Each dot represents one patient. *P*-values and correlation coefficient (*r*) values, as determined by Spearman’s rank correlation test, are shown.

In line with the positive correlation observed between the serum IFN-α and total IgG, IgG1, or IgG4 levels, we found significant positive correlations between IL-33 and total IgG or IgG4 concentrations in the serum of patients with type 1 AIP/IgG4-RD. Once more, no significant correlations between IL-33 and IL-1β, IL-6, TNF-α, IgA, IgE, IgG1, IgG2, or IgG3 were observed (Fig. 4). Collectively, these data suggest that the serum of type 1 AIP/IgG4-RD patients contains higher concentrations of total IgG, IgG1, IgG4, IFN-α, and IL-33, and that the activation of IFN-α and IL-33-mediated signaling pathways is involved in IgG4-biased Ig class switch recombination.

We next explored the relationships between the serum levels of IgG1, IgG4, IFN-α, or IL-33 and the number of affected organs or IgG4-RD RI (Table 1). As shown in Supplementary Fig. S1A, no correlations between the levels of these serum markers and the number of affected organs were observed. In addition, IgG4-RD RI was not positively correlated to any serum marker (Supplementary Fig. S1B).

### Discrimination of type 1 AIP/IgG4-RD from CP using the serum concentrations of IFN-α, IL-33, and IgG4

**Figure 5:**
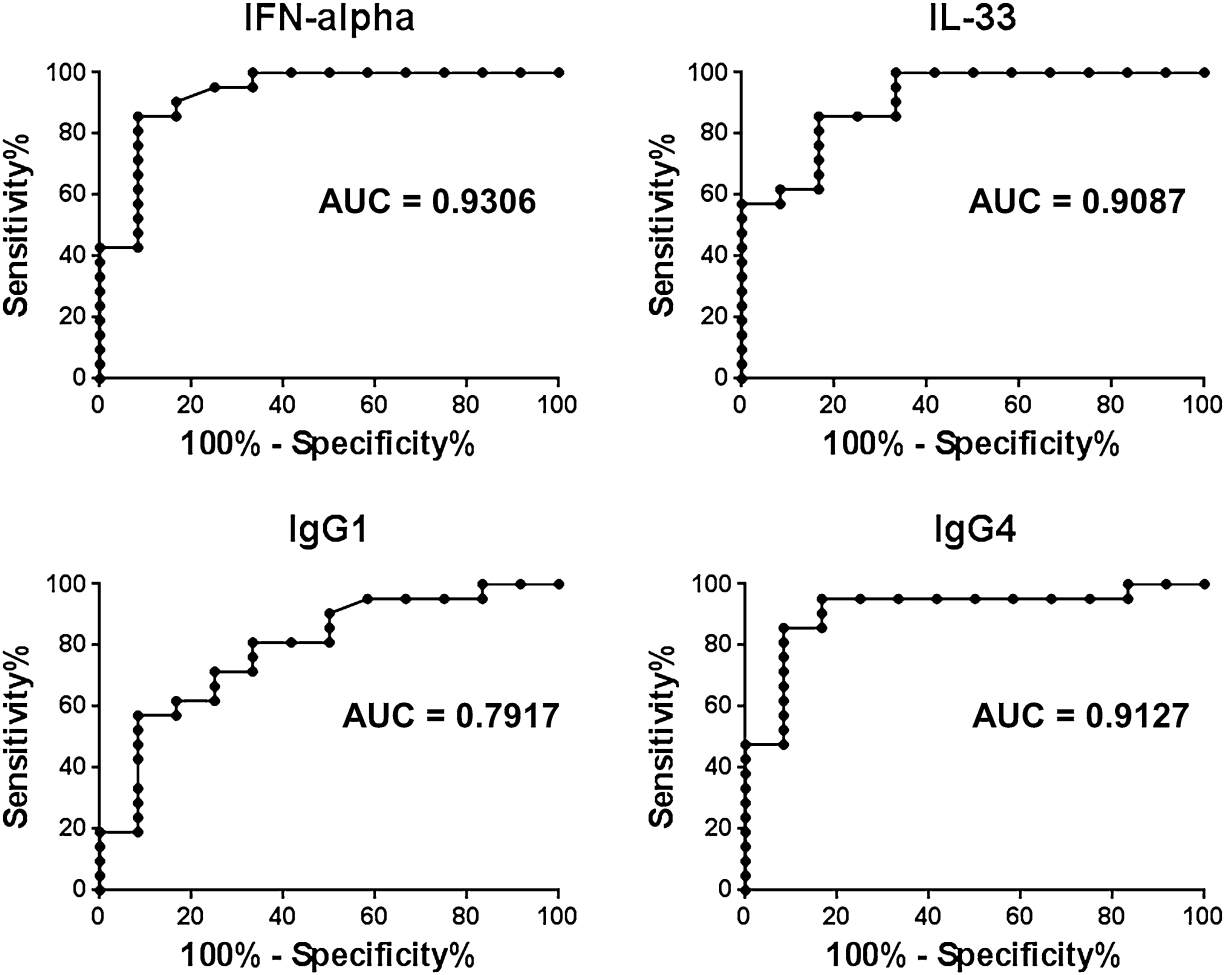
Diagnostic performance of serum IFN-α and IL-33 concentrations for type 1 autoimmune pancreatitis and IgG4-related disease. Receiver operating characteristic curve analysis was performed to assess the utility of serum IFN-α, IL-33, IgG1, and IgG4 levels as biomarkers of type 1 autoimmune pancreatitis and IgG4-related disease. The area under the curve (AUC) was calculated for each biomarker.

We next evaluated the utility of serum IFN-α and IL-33 concentrations as diagnostic biomarkers for type 1 AIP/IgG4-RD. To this end, we performed a ROC curve analysis for the differential diagnosis of type 1 AIP/IgG4-RD and CP and calculated the AUC values for each biomarker. The cut-off values for IFN-α, IL-33, IgG1, and IgG4 were set as 55 pg/mL, 274 pg/mL, 1054 mg/dL, and 182 mg/dL, respectively, as described in the methods section. As shown in Fig. 5 and Table 2, the serum IFN-α concentration discriminated type 1 AIP/IgG4-RD patients from CP patients with an AUC value of 0.9306, a sensitivity of 85.7%, and a specificity of 91.7%. The AUC of IFN-α (0.9306) was higher than that of IL-33 (0.9087), IgG1 (0.7917), or IgG4 (0.9127). No significant difference was observed in the AUC between IgG4 and IFN-α or IL-33. These parameters obtained by ROC curve analysis showed that the serum levels of IFN-α and IL-33 may be useful as diagnostic biomarkers for type 1 AIP/IgG4-RD (in addition to the well-established serum concentration of IgG4).

**Table 2:** Receiver operating characteristic curve analysis.

Variable	AUC	95% CI	*P* value
Serum IgG1	0.7917	0.626–0.958	0.1147*
Serum IgG4	0.9127	0.802–1.024	
Serum IFN-α	0.9306	0.831–1.030	0.8149*
Serum IL-33	0.9087	0.806–1.012	0.9598*

Cut-off point	Sensitivity (%)	Specificity (%)	
Serum IgG1 > 1054 mg/dL	71.4	75	
Serum IgG4 > 182 mg/dL	85.7	91.7	
Serum IFN-α > 55 pg/mL	85.7	91.7	
Serum IL-33 > 274 pg/mL	85.7	83.3	

CI, confidence interval; AUC, area under the curve.

*Statistical differences comparing serum IgG4 AUC and those for serum IgG1, IFN-α, and IL-33.

### Alterations in serum cytokine and IgG subtypes’ responses after prednisolone treatment

**Figure 6:**
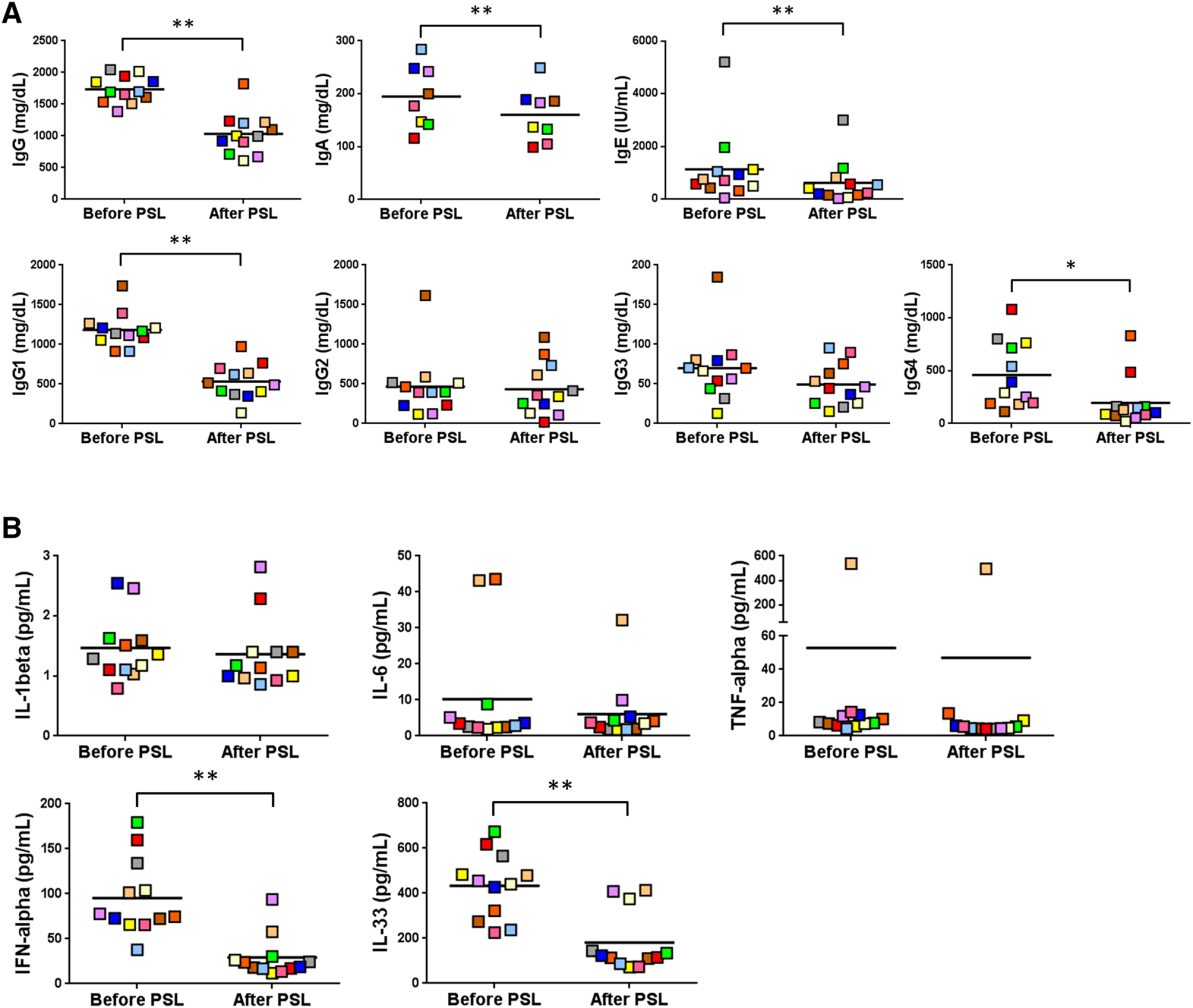
Variation of Ig subtypes’ and innate immunity-related cytokines’ levels in patients with type 1 autoimmune pancreatitis/IgG4-related disease, after prednisolone treatment. Twelve patients with type 1 autoimmune pancreatitis/IgG4-related disease were treated with prednisolone (PSL) and entered in remission. Serum samples before and after PSL treatment were subjected to Ig subtype (**A**) and cytokine (**B**) quantification. Dots of the same color correspond to the values in the same patient before and after PSL treatment. The larger horizontal line indicates the mean value. Statistical significance (before *versus* after PSL treatment) was determined using the Wilcoxon signed rank test: *P < 0.05, **P < 0.01.

Having confirmed the diagnostic utility of serum IFN-α and IL-33 concentrations, we finally addressed whether the levels of these cytokines would reflect disease activity. For this purpose, we measured the serum levels of cytokines and IgG subtypes in 12 patients with type 1 AIP/IgG4-RD, before and after PSL treatment. Oral administration of PSL followed by the scheduled tapering regimen successfully induced clinical remission in all patients. As shown in Fig. 6A and Supplementary Table S2, the induction of remission by PSL treatment decreased the serum concentrations of total IgG, IgG1, IgG4, IgA, and IgE. Marked reductions were especially seen for total IgG, IgG1, and IgG4 levels. In contrast, the serum concentrations of IgG2 and IgG3 were not altered significantly. In addition to the marked reductions in the serum concentrations of total IgG, IgG1, and IgG4, the induction of remission by PSL treatment markedly decreased the serum levels of IFN-α and IL-33. Of note, the levels of prototypical inflammatory cytokines such as IL-1β, IL-6, and TNF-α remained unaffected (Fig. 6B and Supplementary Table S2). Taken together, these data strongly suggest that serum concentrations of IFN-α and IL-33 can be useful biomarkers not only for the diagnosis of type 1 AIP/IgG4-RD, but also for the assessment of disease activity.

## Discussion

In this study, we determined the serum levels of IgG subtypes and innate immunity-related cytokines in patients with type 1 AIP/IgG4-RD. We found that patients with type 1 AIP/IgG4-RD exhibited higher serum concentrations of IgG4, IFN-α, and IL-33, compared with those in patients with CP or HCs. Positive correlations between IgG4, IFN-α, and IL-33 levels were seen in type 1 AIP/IgG4-RD patients. In addition, the induction of remission by PSL markedly lowered the serum concentrations of IgG4, IFN-α, and IL-33. As has been shown in previous reports, including our own, the activation of IFN-α and IL-33 is involved in the immunopathogenesis of experimental AIP and human type 1 AIP/IgG4-RD^1,15–19,30,31^. However, the utility of serum IFN-α and IL-33 levels as biomarkers of type 1 AIP/IgG4-RD has never been evaluated. Here, we provide evidence that measurements of serum IFN-α and IL-33 concentration are useful for the diagnosis and evaluation of disease activity in patients with type 1 AIP/IgG4-RD.

The measurement of IgG4 serum levels is useful for the differential diagnosis between type 1 AIP and pancreatic cancer. It should be noted, however, that a significant fraction of patients with type 1 AIP and pancreatic cancer exhibit normal and elevated levels of this IgG subtype, respectively. Thus, the differential diagnosis between type 1 AIP and pancreatic cancer cannot be exclusively based on the presence or absence of elevated serum IgG4 levels^10,11^. Therefore, to find additional markers for the differential diagnosis between type 1 AIP and pancreatic cancer (perhaps IFN-α and IL-33 serum levels) is essential, and will be the focus of our next study.

We have previously demonstrated that murine experimental AIP and human type 1 AIP/IgG4-RD are characterized by pancreatic accumulation of pDCs, which produce large amounts of IFN-α and IL-33^1,15–19^. Importantly, the development of experimental AIP depends on the activation of signaling pathways triggered by pDC-derived IFN-α and IL-33, since the depletion of pDCs or inhibition of IFN-α or IL-33-mediated signaling pathways effectively prevented the development of experimental AIP^15,16^. In this study, we found that type 1 AIP/IgG4-RD patients exhibited higher serum concentrations of IFN-α and IL-33 than patients with CP or HCs. Thus, patients with type 1 AIP/IgG4-RD are characterized by enhanced IFN-α and IL-33 responses not only in the pancreas but also in the serum. Therefore, it is likely that systemic as well as in situ production of IFN-α and IL-33 might underlie the immunopathogenesis of type 1 AIP and IgG4-RD. In addition, the positive correlations between serum concentrations of these cytokines and IgG4 serum levels reported in this study are fully consistent with the results of our in vitro studies, in which peripheral blood pDCs isolated from type 1 AIP/IgG4-RD patients promoted IgG4 production by HC B cells in an IFN-α-dependent manner^15^. In this study, we have extended these previous data with regard to a possible clinical application by providing evidence that the AUC values of serum IFN-α or IL-33 levels are comparable to those of serum IgG4 AUC values in the ROC curve analysis.

Type 1 AIP/IgG4-RD and systemic lupus erythematosus (SLE) are characterized by elevated serum concentrations of IFN-α^1,32^. In contrast, elevated serum concentrations of IL-33 seen in type 1 AIP/IgG4-RD have not been reported in SLE^33^. IL-33 is a profibrogenic cytokine that induces fibrogenic cytokines such as IL-5 and IL-13^34^. Activation of the IFN-α-IL-33 axis may contribute to the development of a specific type of tissue fibrosis, called storiform fibrosis, in type 1 AIP/IgG4-RD^1–3^ since the inhibition of this axis markedly decreased, not only chronic inflammation, but also pancreatic fibrosis in experimental AIP^15,16,18^. Therefore, the activation of signaling pathways triggered by IL-33 and IFN-α may be involved in the clinical manifestations specific to type 1 AIP and IgG4-RD, but not to SLE. In the future, it will be interesting to determine whether serum concentrations of these two cytokines correlate with the degree of tissue fibrosis in type 1 AIP/IgG4-RD.

Patients with CP and type 1 AIP/IgG4-RD exhibited comparable serum concentrations of the prototypical innate immunity cytokines such as IL-1β, IL-6, and TNF-α. It should be noted, however, that a significant fraction (3/21, 14.3%) of patients with type 1 AIP/IgG4-RD displayed elevated serum concentrations of IL-6 in this study. One concern regarding type 1 AIP/IgG4-RD patients exhibiting elevated IL-6 levels might be the simultaneous occurrence of type 1 AIP/IgG4-RD and MCD. Indeed, it has been reported that differentiation between MCD and IgG4-RD may be difficult in some cases^35–37^. Moreover, clinical cases with overlapping IgG4-RD and MCD were also described^38,39^. As for the clinical manifestations, Tsukuda et al. reported that elevated serum levels of IL-6 are associated with the involvement of the bile duct, spleen, and liver in IgG4-RD patients^40^. Although we could not confirm the association between serum IL-6 concentrations and specific clinical manifestations, probably due to a relatively small number of patients in this study, further studies are required to sub-classify the MCD-like subtype within type 1 AIP/IgG4-RD patients based on the evaluation of serum IL-6 levels.

Our serum IgG subtype analysis revealed higher serum concentrations of IgG1 and IgG4 in type 1 AIP/IgG4-RD patients than in CP patients. Importantly, serum levels of both IgG subtypes were considerably reduced by PSL treatment. Thus, our results suggest that both IgG1 and IgG4 faithfully reflect disease progression. It should be noted, however, that the subtype of IgG causing chronic fibroinflammatory responses has not been identified in type 1 AIP/IgG4-RD^1–3^. IgG1, which activates the complement system and Fcγ receptors play pathogenic roles in various autoimmune and infectious diseases^41,42^. In contrast, IgG4 induces anti- rather than pro-inflammatory responses as this IgG subtype lacks the ability to activate the complement and Fcγ receptors^41,42^. Therefore, it is likely that an enhanced IgG4 response is an epiphenomenon associated with chronic inflammation in type 1 AIP/IgG4-RD. This idea is supported by translational studies in which IgG1 isolated from the serum of type 1 AIP patients was found to cause more severe pancreatic injury than IgG4 upon intraperitoneal injection into neonatal mice^43^. Moreover, the injection of IgG4 attenuated pancreatic injury induced by the injection of IgG1^43^. In line with these findings, two recent studies that identified candidate autoantigens in type 1 AIP and IgG4-RD provided evidence that the IgG4 subtype inhibits the binding of the IgG1 subtype to the autoantigen epitopes^44,45^. These reports suggested pathogenic roles for the IgG1 rather than the IgG4 subtype. Given the fact that relapse sometimes occurs even in type 1 AIP/IgG4-RD patients with normal IgG4 concentrations^12^, monitoring serum concentrations of both IgG1 and IgG4 might be necessary in this autoimmune disorder.

In contrast to the determined IgG1 and IgG4 levels, we did not detect any significant elevation of serum IgG2 or IgG3 concentrations in patients with type 1 AIP/IgG4-RD. Recently, Chan et al. reported significantly elevated serum IgG2 levels in patients with IgG4-RD^46^. This discrepancy can be partially explained by the difference in the distribution of affected organs in IgG4-RD. Chan’s study examined serum IgG2 concentrations in 43 patients with orbital IgG4-RD, including two type 1 AIP cases^46^. Our study examined serum IgG subtypes in 21 type 1 AIP/IgG4-RD patients that did not present eye involvement. Whether IgG subtype responses depend on the distribution of affected organs requires further investigation.

Although the data presented in this study support the utility of serum IFN-α and IL-33 as biomarkers of type 1 AIP/IgG4-RD, our study had several limitations. First, this retrospective study was conducted with patient samples collected in a single center. Future multicenter studies addressing the utility of these cytokine markers in a prospective manner are required to confirm our data. Secondly, most patients enrolled in this study were type 1 AIP patients, and thus, abnormal innate immune responses in the pancreas might have some influence on their serum cytokine responses. Given the fact that IgG4-RD affects almost all the organs in the body, it is possible that serum cytokine responses may differ depending on the predominantly affected ones. Therefore, serum cytokine responses need to be evaluated in various types of IgG4-RD, such as IgG4-related sialadenitis, IgG4-related lung disease, and IgG4-related kidney disease, in addition to type 1 AIP^1–3^.

In conclusion, we found that the serum of patients with type 1 AIP/IgG4-RD showed high concentrations of IFN-α and IL-33, which, in turn, positively correlated with the serum IgG4 levels. Furthermore, their levels were markedly decreased following PSL treatment. Therefore, we conclude that the serum concentrations of IFN-α and IL-33 may serve as biomarkers for the diagnosis of type 1 AIP/IgG4-RD, as well as for monitoring purposes.

## Supplementary information

Supplementary Information.
