# Supplementary material for: Identification of serum IFN-α and IL-33 as novel biomarkers for type 1 autoimmune pancreatitis and IgG4-related disease
Source: Sci Rep. 2020 Sep 16;10:14879. doi: 10.1038/s41598-020-71848-4 (PMC7495433; doi:10.1038/s41598-020-71848-4)
Supplement: Supplementary file 1 — Supplementary Information. [file 41598_2020_71848_MOESM1_ESM.docx]

**Identification of serum IFN-α and IL-33 as novel biomarkers for type 1 autoimmune pancreatitis and IgG4-related disease**

Kosuke Minaga^1^, Tomohiro Watanabe^1^, Akane Hara^1^, Ken Kamata^1^, Shunsuke Omoto^1^, Atsushi Nakai^1^, Yasuo Otsuka^1^, Ikue Sekai^1^, Tomoe Yoshikawa^1^, Kentaro Yamao^1^, Mamoru Takenaka^1^, Yasutaka Chiba^2^, Masatoshi Kudo^1^

^1^Department of Gastroenterology and Hepatology, Kindai University Faculty of Medicine, Osaka-Sayama, Osaka, Japan

^2^Clinical Research Center, Kindai University Hospital, Osaka-Sayama, Osaka, Japan

**SUPPLEMENTARY DATA**

**Supplementary Table S1**. Statistical data regarding serum Igs and cytokines quantification (mean ± standard deviation and *P* values)

|  | **HCs** | **CP** | **type 1 AIP/IgG4-RD** |
| --- | --- | --- | --- |
| Serum IgG | 1167 ± 269.7 | 1346 ± 309.4 | 1810 ± 413 |
| Serum IgA | 261.0 ± 60.74 | 325.3 ± 103.7 | 256.5 ± 137.0 |
| Serum IgE | 203.0 ± 312.1 | 459.4 ± 474.2 | 818.5 ± 1120 |
| Serum IgG1 | 513.8 ± 295.9 | 741.8 ± 405.8 | 1189 ± 447.7 |
| Serum IgG2 | 613.6 ± 278.5 | 831.6 ± 422.1 | 473.9 ± 365.8 |
| Serum IgG3 | 71.11 ± 61.65 | 52.62 ± 36.01 | 74.27 ± 43.66 |
| Serum IgG4 | 38.60 ± 26.68 | 105.2 ± 114.5 | 530.2 ± 483.2 |
| Serum IL-1β | 1.254 ± 0.506 | 1.791 ± 0.963 | 1.340 ± 0.441 |
| Serum IL-6 | 2.977 ± 1.592 | 6.357 ± 3.275 | 8.767 ± 13.15 |
| Serum TNF-α | 5.621 ± 1.905 | 9.492 ± 8.862 | 35.84 ± 115.7 |
| Serum IFN-α | 3.592 ± 3.475 | 29.64 ± 20.71 | 92.53 ± 40.74 |
| Serum IL-33 | 46.38 ± 35.09 | 203.8 ± 102.5 | 454.3 ± 176.4 |

|  | ***P v*alue*** | | | ***P* value**** |
| --- | --- | --- | --- | --- |
|  | **HCs vs. CP** | **HCs vs. type 1 AIP/IgG4-RD** | **CP vs. type 1 AIP/IgG4-RD** |  |
| Serum IgG | NS | 0.0006 | 0.006 | 0.0003 |
| Serum IgA | NS | NS | NS | NS |
| Serum IgE | NS | 0.0456 | NS | NS |
| Serum IgG1 | NS | 0.0009 | 0.0147 | 0.0005 |
| Serum IgG2 | NS | NS | 0.0414 | 0.0302 |
| Serum IgG3 | NS | NS | NS | NS |
| Serum IgG4 | NS | <0.0001 | <0.0001 | <0.0001 |
| Serum IL-1β | NS | NS | NS | NS |
| Serum IL-6 | 0.0156 | NS | NS | 0.0362 |
| Serum TNF-α | NS | 0.033 | NS | 0.0305 |
| Serum IFN-α | <0.0001 | <0.0001 | <0.0001 | <0.0001 |
| Serum IL-33 | <0.0001 | <0.0001 | <0.0001 | <0.0001 |

*Bonferroni corrected Mann-Whitney U test was used for the comparison of two groups. **Kruskal-Wallis test was used for the comparison of three groups.

HCs, healthy controls; CP, chronic pancreatitis; type 1 AIP/IgG4-RD, type 1 autoimmune pancreatitis/IgG4-related disease; NS, not significant.

**Supplementary Table S2**. Statistical data regarding serum Igs and cytokines quantification, before and after prednisolone treatment (mean ± standard deviation of differences and *P* values)

|  | **mean ± standard deviation of differences** | ***P* value*** |
| --- | --- | --- |
| Serum IgG | -700.4 ± 426.3 | 0.0010 |
| Serum IgA | -34.38 ± 25.59 | 0.0078 |
| Serum IgE | -519.1 ± 608.2 | 0.0034 |
| Serum IgG1 | -651.8 ± 346.2 | 0.0010 |
| Serum IgG2 | -33.88 ± 273.4 | NS |
| Serum IgG3 | -20.35 ± 37.23 | NS |
| Serum IgG4 | -264.4 ± 364.1 | 0.0210 |
| Serum IL-1β | -0.103 ± 0.637 | NS |
| Serum IL-6 | -4.107 ± 11.80 | NS |
| Serum TNF-α | -6.017 ± 12.05 | NS |
| Serum IFN-α | -66.08 ± 47.60 | 0.0010 |
| Serum IL-33 | -252.5 ± 176.6 | 0.0005 |

*Statistical significance was determined by the Wilcoxon signed rank test (before *versus* after prednisolone treatment).

**Supplementary Results**

**Correlation between serum levels of IgG1, IgG4, IFN-α, or IL-33 and the number of affected organs**

In cases with type 1 autoimmune pancreatitis (AIP) and IgG4-related disease (IgG4-RD), serum IgG4 level over 280 mg/dL is reported to be associated with multiple organ involvement and the risk of relapse^1,2^. In addition, IgG4-RD responder index (IgG4-RD RI), which is a scoring system using the serum IgG4 level and disease activity of affected organs, is also used for evaluation of disease activity^3^. We explored the relationship between the serum levels of IgG1, IgG4, interferon (IFN)-α, or interleukin (IL)-33 and the number of affected organs or IgG4-RD RI in 21 patients with type 1 AIP/IgG4-RD enrolled in this study (Supplementary Figure S1). The number of affected organs was determined by positron emission tomography/computed tomography (PET/CT) using ^18^F-2-fluoro-2-deoxy-D-glucose in 19 patients and contrast-enhanced whole- body CT in two patients. Serum concentrations of IgG1, IgG4, IFN-α, and IL-33 were measured at the time of disease diagnosis in all cases. As shown in Supplementary Fig. S1A, no correlations between the levels of these serum markers and the number of affected organs were observed. In addition, IgG4-RD RI was not positively correlated to any serum marker (Supplementary Fig. S1B).

**Supplementary References**

1 Culver, E. L. *et al.* Elevated Serum IgG4 Levels in Diagnosis, Treatment Response, Organ Involvement, and Relapse in a Prospective IgG4-Related Disease UK Cohort. *Am J Gastroenterol* **111**, 733-743, doi:10.1038/ajg.2016.40 (2016).

2 Tang, J., Cai, S., Ye, C. & Dong, L. Biomarkers in IgG4-related disease: A systematic review. *Semin Arthritis Rheum* **50**, 354-359, doi:10.1016/j.semarthrit.2019.06.018 (2020).

3 Carruthers, M. N., Stone, J. H., Deshpande, V. & Khosroshahi, A. Development of an IgG4-RD Responder Index. *Int J Rheumatol* **2012**, 259408, doi:10.1155/2012/259408 (2012).


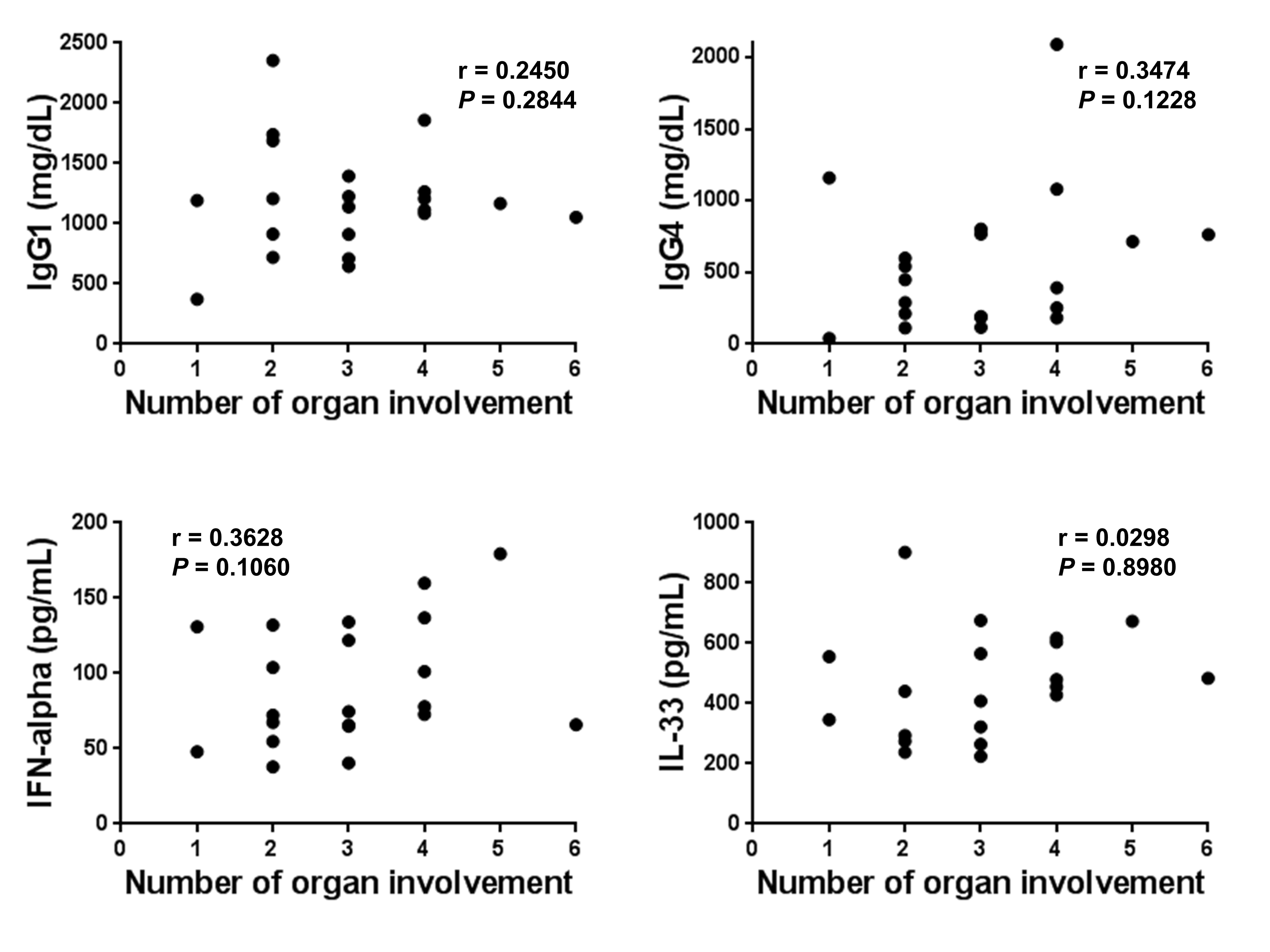
 **(A)**


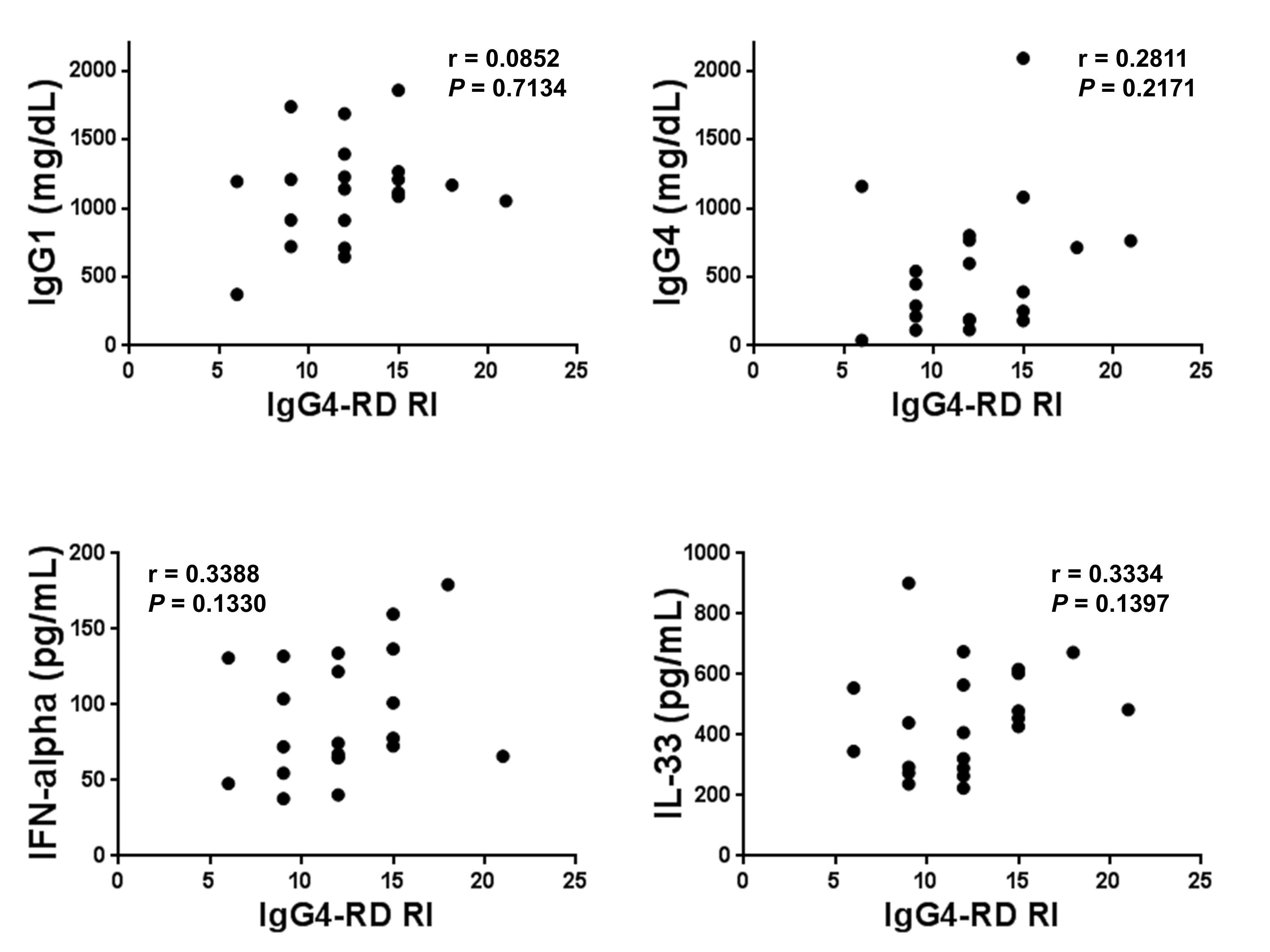
**(B)**

**Supplementary Figure S1. Lack of correlation between the number of affected organs or IgG4-related disease responder index and serum concentrations of IgG1, IgG4, IFN-α, and IL-33.** Serum concentrations of IFN-α, IL-33, IgG1, and IgG4 were measured in 21 patients with type 1 autoimmune pancreatitis (AIP)/IgG4-related disease (IgG4-RD). **(A)** The number of organs involved was determined by positron emission tomography/computed tomography in 19 patients and contrast-enhanced computed tomography in two patients and correlated with the above mentioned cytokines/Ig serum levels. **(B)** IgG4-RD responder index (IgG4-RD RI) was calculated in each patient and correlated with the above mentioned cytokines/Ig serum levels. Each dot represents one patient. *P*-values and correlation coefficient (*r*) values, as determined by Spearman’s rank correlation test, are shown.
